# Parental Psychological Control: Maternal, Adolescent, and Contextual Predictors

**DOI:** 10.3389/fpsyg.2021.712087

**Published:** 2021-09-21

**Authors:** J. Carola Pérez, Paula Huerta, Bernardita Rubio, Olga Fernández

**Affiliations:** ^1^Centro de Apego y Regulación Emocional, Universidad del Desarrollo, Santiago, Chile; ^2^Master’s Program in Adolescent Psychology, Universidad del Desarrollo, Santiago, Chile; ^3^Psychiatry and Mental Health Department, Faculty of Medicine, Universidad de Chile, Santiago, Chile

**Keywords:** maternal psychological control, adolescent irritability, adolescent effortful control, maternal separation anxiety, inter-parental conflict

## Abstract

Parental psychological control (PC) hinders the development of autonomy, identity formation, and the attainment of self-determination and individuation of adolescents. The aim of this study was to deepen the understanding of which conditions increase the risk of the use of maternal PC by simultaneously considering the contribution of adolescent temperament, maternal separation anxiety, and adolescents’ perception of interparental conflict. A correlational study involving a sample of 106 Chilean adolescent-mother dyads was done. Adolescents were, on average, 15.42 years old (SD = 1.09) and 77% male. Mothers were, on average, 45.46 years old (SD = 6.39). We administered self-report questionnaires to the adolescent measuring effortful control and frustration as temperamental dimensions, along with the perception of interparental conflict. Mothers reported on their separation anxiety. Both the adolescents and their mothers reported on the use of maternal PC. Adolescents reported higher levels of maternal PC than their mothers did. All predictors were associated with PC reports. Higher levels of maternal anxiety about adolescent distancing, inter-parental conflict, and adolescent frustration were associated with higher reported levels of PC. In contrast, higher levels of adolescent effortful control were associated with lower levels of maternal PC. Finally, when maternal separation anxiety and inter-parental conflict were high there was a higher use of maternal PC. The present findings inform on how adolescent’s self-regulatory skills could reduce the risk of being exposed to maternal PC. And highlight the importance of using a systemic and interactional conceptualization when trying to understand their use.

## Introduction

Psychology research has focused on parental practices for many decades, due to the impact these have on children’s emotional, cognitive, and social development (Schaefer, 1965; Maccoby and Martin, 1983; Barber, 1996; Barber and Harmon, 2002; Aunola and Nurmi, 2005). Psychological control (PC) is a parental practice characterized by intrusive and manipulative behaviors aimed at children or adolescents’ thoughts and feelings, through which adults exert power by controlling their children’s psychological world. These behaviors include guilt induction, affection withdrawal, and/or the manipulation of the parent–child relationship (Barber, 2002; Barber and Harmon, 2002). Parents who use this type of control are characterized by an inability to differentiate their own needs from those of their children; even more so, they fail to visualize their children or adolescents’ point of view (Barber and Harmon, 2002).

Regarding the effects of parental PC on adolescent well-being and adjustment, authors such as Barber and Harmon (2002), Soenens and Vansteenkiste (2010), and Scharf and Goldner (2018) provide extensive evidence on how this type of parental control hinders the development of autonomy, identity formation, and the attainment of self-determination and individuation. Parental PC has shown negative consequences in adolescents’ emotional adjustment (Gugliandolo et al., 2015; Ha and Jue, 2018), which in turn is related to various psychological problems, such as low self-esteem, poor adjustment, and internalizing and externalizing disorders. Parental PC has shown detrimental effects on child development in multiple cultural settings (Pettit et al., 2001; Barber et al., 2005; Finkenauer et al., 2005; Koçak et al., 2017; Symeou and Georgiou, 2017; Scharf and Goldner, 2018) including collectivist societies which perceive parental control as an expression of love and care (Gargurevich and Soenens, 2016). In Chile, parental PC has been scarcely studied, but the existing research results are consistent with the notion that PC has a negative effect on adolescent wellbeing (Casassus Rodino et al., 2011; Pérez and Cumsille, 2012).

The evidence available on the negative effects of PC on adolescents, highlights the need for identifying and understanding the conditions that could increase its use and underlying mechanisms associated with these parental practices. Belsky’s (1984) model has been used to understand and explain why parents use PC and the consequences this parental practice produces (Barber et al., 2002; Scharf and Goldner, 2018). Belsky’s model considers the influence of adult (parental) characteristics, adolescent characteristics, and contextual-related factors.

The maternal characteristics associated to an increase use of maternal PC toward their children are competence-related personality features (e.g., low self-worth and perfectionism) and autonomy-related difficulties (e.g., separation anxiety) (Soenens et al., 2005, 2006; Laird, 2011; Scharf and Goldner, 2018). In the present study, we focus on maternal separation anxiety, which refers to feelings or unpleasant emotional states (such as anxiety, sadness, and frustration) associated with the adolescents’ independence and autonomy, such as, adolescents’ affective distancing of their families and decreased involvement with them, and their progressive involvement with people outside of the family (Hock et al., 2001). Adults with parental separation anxiety, may express this type of anxiety through the use of PC, such as guilt induction and conditional child’s approval. Through using these PC strategies, these parents are attempting to maintain physical closeness and close adolescent-parent emotional ties. Soenens et al. (2010) called this strategy dependency-oriented psychological control (DPC). DPC might mediate the relationship between separation anxiety reported by parents and the emerging adults’ pathology of the separation-individuation process. Nevertheless, this mediating process would be especially relevant in the case of mothers, who report higher levels of DPC when compared to fathers (Kins et al., 2011). In Latin American and Chilean culture, it is plausible to hypothesize that separation anxiety toward their children would be higher in mothers than fathers since child rearing is still mainly perceived as a maternal responsibility (Aguayo et al., 2011). Based on these cultural expectations, psychologists expect to see greater separation anxiety in mothers than in fathers (Minuchin, 1986; Montesinos, 2007). A previous study shows the importance attributed to motherhood in Chilean women’s life project, and the prescriptive nature of mythology on motherhood sustained by them concerning the care and responsibility for the children (Pérez and Jaramillo, 2009). In fact, commitment to feminine-maternal identities in Chilean mothers could transform normative adolescent autonomy into a threat to the maternal role, leading to a potential increase of control toward their adolescent children.

Regarding the child’s or adolescent’s characteristics, additionally to sociodemographic characteristics such as gender and age, difficult temperament (dysregulated or inhibited) and internalizing/externalizing symptoms are important predictors of maternal PC (Rubin et al., 1997; Pettit et al., 2001; Scharf and Goldner, 2018). Studies have shown that difficult child temperament, characterized by low positivity or high negative emotionality, are associated with maternal PC in different cultural settings such as United States, Canada, and Finland (LaFrenière and Dumas, 1992; Walling et al., 2007; Laukkanen et al., 2014) and Chile (Pérez and Cumsille, 2012).

Temperament is defined as individual differences in reactivity and self-regulation (Rothbart and Bates, 2006). Reactivity refers to physiological and behavioral systems responsiveness, while self-regulation refers to the functioning of neural and behavioral processes that modulate this reactivity (Henderson and Wachs, 2007). Rothbart and Bates (2006) proposed a psycho-biological model of temperament that establishes three dimensions: (a) surgency, individual’s level of activity and his/her tendency to seek novel situations; (b) negative affect, a person’s propensity to experience negative emotions such as discomfort, fear, dysphoria, anger, and/or frustration; and (c) effortful control, the ability to suppress a dominant response and replace it with a subdominant response, reflecting the presence of a self-regulatory mechanism.

Temperament influences adolescents’ adjustment and/or maladjustment in various ways. For example, these traits increase or minimize the adolescents’ responses to environmental events, bias their processing of information, or evoke reactions in the people around them (Rothbart, 2004). Shiner and Caspi (2003) have labeled this evocation phenomenon environmental elicitation. Given that adolescents’ temperamental characteristics can generate different reactions in their environment, thus influencing the way in which other people (e.g., their parents) interact with and react toward them. In fact, most scholars accept the idea that children and adolescents with some specific characteristics, like difficult temperament, are more challenging for parenting (Sanson et al., 2004; Paulussen-Hoogeboom et al., 2007).

Finally, regarding the contextual-related factors of Belsky’s (1984) model, culture (Chen et al., 2016), stressful events or environments, contextual hardship and inter-parental relationships, have shown to impact the use of parental PC (Scharf and Goldner, 2018). In the present study, we focus on inter-parental conflict, because it has been associated to elevated levels of stress in parents (Buehler et al., 2006; Huth-Bocks and Hughes, 2008; Neff and Karney, 2009), and both, a stressful environment and inter-parental conflict are variables associated to high levels of maternal PC (Stone et al., 2002; Krishnakumar et al., 2003; Cabrera et al., 2006; Koçak et al., 2017).

The present study aims to deepen the understanding of the conditions that promote the use of maternal PC toward their adolescent children. To do so, it analyzes whether maternal PC – as reported by mothers and by adolescents – is linked to mothers’ separation anxiety, to adolescents’ effortful control and frustration, and inter-marital conflict as experienced by the adolescent. It also seeks to offer information to better understand how these variables interact, and identify conditions that increase the use of maternal PC. Few studies have jointly considered the three sources (Laird, 2011) that can influence the use of PC, most of them consider the characteristics of adolescents, parents or the context, or a combination of two of these sources (Pettit et al., 2001; Walling et al., 2007). Therefore, this research advances in the understanding of the use of maternal PC, by considering the joint effect exerted by multiple domains of influence.

One of the methodological challenges we face when studying joint effects on maternal PC, is the potential multicollinearity between predictors. For example, Laird (2011) who addressed a similar research question to ours, reported that maternal separation anxiety is a predictor of mother-reported PC, but not maladaptive perfectionism when adults’, adolescent’ and contextual variables are included in the model.

Considering these challenges, for the effects of the present study, among the relevant parental characteristics we chose only to include the mothers’ separation anxiety as a proxy of parental autonomy-related difficulties. Among the adolescents’ characteristics, only adolescents’ effortful control and frustration were included, because they are core aspects that capture central elements of temperamental characteristics, which have also been identified as risk factors for the development of psychopathology or poor psychosocial functioning (Oldehinkel et al., 2004; Oldehinkel et al., 2007; Baetens et al., 2011; Muris et al., 2011). Finally, regarding the contextual characteristics that influence maternal PC, considering how inter-marital conflict is a source of stress for members of the parental dyad, and that the influence of inter-marital conflicts varies according to by who in the family system reports it (Grych et al., 1992; Iraurgi et al., 2008), showing a greater impact in adolescent behavior when it is reported by the adolescent (Grych et al., 1992; Kitzmann and Cohen, 2003). In the present study, we will only measure inter-parental conflict as a contextual variable from the adolescents’ perspective.

In line with Belsky’s (1984) model, we hypothesize that maternal separation anxiety, adolescent temperamental dimensions, and intra-marital conflict are associated with associated with maternal PC, each of them influencing the use of this type of parental control. Specifically, we expect that higher levels of maternal anxiety about adolescent distancing, inter-parental conflict, and adolescent frustration corresponded with higher reported levels of maternal PC. We also expect a higher level of adolescent effortful control associated with lower levels of maternal PC.

Based on the fact that adolescents with temperamental characteristics of high frustration and reduced effortful control, have a higher risk of being exposed to maternal PC due to being less capable of self-regulating their behaviors (Van Beveren et al., 2020). The joint effect of both temperamental dimensions on the use of maternal PC will be explored.

Additionally, the coexistence of high levels of inter-parental conflict and maternal separation anxiety could increase the use of maternal PC. This condition would involve high levels of maternal stress (Sturge-Apple et al., 2003) that might decrease self-regulatory capacities (Muraven and Baumeister, 2000) and increase the use of maternal PC. Likewise, mothers might use control strategies toward others to reduce their own anxiety (Lyons-Ruth and Jacobvitz, 2016).

Finally, the present study considers a multi-informant perspective, collecting both adolescents’ and mothers’ reports of maternal PC. By doing so it addresses one of the limitations stipulated in previous studies (Gugliandolo et al., 2015; Gargurevich and Soenens, 2016; Scharf and Goldner, 2018). While Korelitz and Garber (2016) indicated that children report higher levels of PC than their mothers, we do not propose a specific hypotheses on how who reports the maternal PC will influence the impact of adolescents’, mothers’ or contextual characteristics on the use of maternal PC, based on limited previous evidence (Pettit et al., 2001; Walling et al., 2007; Laird, 2011).

## Materials and Methods

### Participants

A total of 141 Chilean mothers accepted to participate in the study, but only 106 of their adolescent children. So, the final sample included 106 Chilean mother–adolescent dyads (14–18 years old) that resided in the same home. This was a non-probabilistic sample.

Seventy-seven percent were mother–son dyads. The mothers’ average age was 45.46 years (SD = 6.39), the sons’ average age was 15.35 years old (SD = 1.07), and the daughters’ average age was 15.63 years old (SD = 1.17). When comparing the mother–son to the mother–daughter dyads, we observed no differences in adolescents’ age, *t*(104) = −1.07, *p* = 0.29; however, the sons’ mothers were older (*M* = 46.15, SD = 6.10) than the daughters’ (*M* = 36.13, SD = 6.92, *t*(104) = 2.07, *p* = 0.04).

Sixty-eight percent of the surveyed adolescents lived with both parents, 24% lived only with their mother (92% of which were separated or divorced mothers), and 8.5% lived with their mother and a stepfather. Consistent with this data, 32% of the adolescents reported that their parents were divorced/separated. When comparing the mother–son to the mother–daughter dyads, we observed no differences regarding the persons with whom the adolescents lived, χ^2^(2, *N* = 106) = 1.42, *p* = 0.49; or whether their parents were divorced/separated, χ^2^(2, 106) = 4.05, *p* = 0.13.

### Instruments

#### Psychological Control-Disrespect Scale

Based on a qualitative study on adolescents’ notion of PC, Barber et al. (2012) developed a self-report scale of perceived parental PC. Pérez and Cumsille (2012) translated the scale into Spanish and administered it to a Chilean sample of adolescents. In their Exploratory Factor Analysis, they retained a solution of one-factor that includes seven of the eight original items; they excluded the item “my mother expects too much of me” based on its low factorial loading. The low factor loading of this item was also reported by Barber et al. (2012) when adolescents of Costa Rica and South African-Black participants were included in their sample. In the present study, adolescents and their mothers completed the scale. Samples of the items includes the following: “My mother embarrasses me in public” (adolescent version) and “I embarrass my son/daughter” (mother version); and “She tries to make me feel guilty for something I did or didn’t do” (adolescent version) and “I try to make my son/daughter feel guilty.” (Mother’s version). A multi-group Confirmatory Factor Analysis (CFA) was done. It indicated that a one-factor solution was metric invariance in mother and adolescent maternal PC scale Δχ^2^(6 *df.*, *N* = 106) = 2.391, *p* = ,878 (see Supplementary Table S1). On a 5-point Likert scale ranging from never (1) to always (5), adolescents reported how often their parents displayed the behaviors included in the scale, and mothers reported how often they engaged in such behaviors. We scored each scale by averaging the scores of the items as reported by the adolescents (α = 0.73) and by their mothers (α = 0.77).

#### Maternal Separation Anxiety

We used the subscale “Anxiety about adolescent distancing,” from the self-report scale “Parents of Adolescents Separation Anxiety Scale” (PASAS), to measure maternal separation anxiety. This subscale reflects the feelings of discomfort and/or loss that parents experience due to the increased relationships between their adolescent child and parties outside the family context, their reduced involvement as parents with their adolescent child, and the fact that they spend less time doing joint activities (Hock et al., 2001). In order to use this instrument in the present study, according to the recommendation to adapt the scales culturally (Muñiz et al., 2013), we translated into Spanish the original PASAS scale and then back-translated into English. A CFA considering all mothers who accepted participate in the study indicated that the original two-factor solution did not fit with the data, χ^2^(559 *df., N* = 141) = 1060.908, *p* < 0.001, RMSEA = 0.080 (95% CI 0.072–0.087), CFI = 0.73. Nevertheless, all items of maternal separation anxiety loaded significantly on one of the factors modeled. Based on this information, an Exploratory Factor Analysis was done. A two-factor and three-factor related solutions were plausible. A three-factor solution was selected based on empirical and theoretical points of view. The factor-1 represent the Maternal Separation Anxiety dimension, including 20 of the 21 items from the original “anxiety about adolescent distancing” subscale plus items 9 (My teenager is a source of comfort for me when I’m upset) and 14 (I will miss seeing my teenager’s belongings around the house after he/she leaves home), for which factor loadings were significant in the Chilean sample (see Supplementary Table S2). Mothers indicated their degree of agreement with statements that reflect how they felt at the time of the survey or how they thought they would feel in the near future, as their child grows older, on a 5-point Likert scale ranging from strongly disagree (1) to strongly agree (5). Examples of items included are as follows: “I feel most content when I know my child is sleeping under my roof” and “I really miss holding my teenager like I did when he/she was younger.” We averaged the items, and their reliability was α = 0.91.

#### Inter-Parental Conflict From the Children’s Perspective

We used the “Conflict Properties” subscale of the self-report scale Inter-Parental Conflict from the Children’s Perspective (CPIC; Iraurgi et al., 2008). Iraurgi et al. (2008) culturally adapted the scale developed by Grych et al. (1992). The subscale (16 items) used in this study, reflects children’s perception of the frequency, intensity, and stability of interparental conflicts, as well as of the difficulties involved in solving them. The adolescents needed to express if each item describes their parents’ arguments and how they felt in these situations, on a scale from zero (false) to two (almost true) (Iraurgi et al., 2008). We added the items up, and their reliability was α = 0.92.

It is important mentioning that 92% of the families in which the adolescent lived only with his or her mother, corresponded to separated or divorced mothers. Regarding the possible impact that parents not living together could have on the measurement of inter-parent conflict from the adolescent’s perspective, it is necessary to consider that not living with their father does not mean that the mothers and/or adolescents had no relationship with him. Also, inter-parental conflict is not uncommon among divorced/separated parents (Lansford, 2009; van Dijk et al., 2020). Thus, to establish the possible impact of parents not living together, it was analyzed whether there were differences in the maternal PC reported by the mother and adolescent according to the types of family. No significant differences were found in the level of maternal PC when mothers, *F*(2,103) = 0.51, *p* = 0.60, and adolescents report it, *F*(2,103) = 2.07, *p* = 0.13, according to family type. Based on these results no additional adjustment was done to the data analysis plan.

#### Adolescent Temperament

We used the Effortful Control and Frustration scales of the Early Adolescent Temperament Questionnaire-Revised (Ellis and Rothbart, 2001) adapted to Chilean samples (Hoffmann et al., 2017). The adolescents reported the degree to which the items in the questionnaire described them, using a 5-point Likert scale (1 = Does not describe me adequately to 5 = Describes me very well). The Frustration dimension of the Negative Affect factor accounts for negative affect related to the interruption of ongoing tasks or goal blocking (e.g., items: “I get very frustrated when I make a mistake in my school work”). We averaged the items and found the nine items have a reliability of α = 0.69. We considered the items of the three subscales of the Effortful Control factor: “I finish my homework before the due date” (Activational control); “When interrupted or distracted, I forget what I was about to say” (Attention, reversed item); and “When someone tells me to stop doing something, it is easy for me to stop” (Inhibitory control). We averaged all items to calculate a single Effortful Control score (17 items, α = 0.71).

### Procedure

We obtained the sample from five schools located in Santiago, Chile. This data was collected before COVID-19 pandemic. The research team invited mothers to participate in the present study and to authorize their children to be part of it. Two strategies were used to contact mothers. In most cases, research team members attended periodic parent meetings at schools where they presented the research objectives and methodology. Based on the estimated universe of mothers from the contacted classes, the acceptance rate was approximately 35%. The second strategy was snowball sampling, mothers contacted through this strategy were invited to group meetings in which a member of the research team informed them about the study. Mothers who agreed to participate signed the Informed Consent and completed their questionnaires. Afterward, we invited the authorized adolescents to participate. The adolescents who agreed to participate signed their Informed Assent and completed their questionnaires individually in their classrooms. Twenty-one percent of the authorized adolescents decided not to participate in the study. The participants did not receive any compensation for participating in the study.

### Ethical Approval

This study was developed within the context of the FONDECYT 11130041 project, which was approved by the Ethics Committee of the Universidad del Desarrollo. All procedures performed in studies involving human participants were in accordance with the ethical standards of the Universidad del Desarrollo and with the 1964 Helsinki declaration and its later amendments or comparable ethical standards.

### Data Analysis

In the first place, we descriptively analyzed the data and estimated the Pearson correlations between variables.

From a theoretical point of view, it is reasonable to propose that adolescents- and mothers-view on aspects of their relationship (such as PC) are related. It would be expected some degree of data non-independence, based on their kinship linkage or their quotidianly interaction (Kenny et al., 2006). In fact, Korelitz and Garber (2016) meta-analysis indicates a correlation of 0.27 in the reports of PC between adolescents and their mothers. Empirically, a single-level model (that means independent data) respect to a two-level model (informant as Level-1 and mother-adolescent dyads as Level-2) were compared by using ML-2ΔLL test = 2.5 (1 *df*.), *p* = 0.11 (Hoffman, 2015), which indicated that the single-level was more adequate. Nevertheless, based on the actual power of the study to test the non-independence of the data (<0.60), we assume a “liberal alpha value (0.20)” as Kenny et al. (2006, p. 50) recommend.

So, in order to test the hypotheses, we conducted a Hierarchical Linear Model (MLR estimation method) on the PCs the adults and adolescents reported. SPSS v.23 and HLM software were used. A model-building approach was used (Hoffman, 2015). In the Null Model, the Intra-Class correlation was estimated. In the first model, we included “Informant” as a Level-1 predictor to account for the differences in the PCs the adolescents and their mothers reported. In the second model, we included dyad type (mother–son vs. mother–daughter) as a control variable (because the adolescents’ sex was related to the mother-reported PC), and we included maternal separation anxiety, inter-parental conflict and both dimensions of adolescents’ temperament as Level-2 predictors (all mean-centered). In the third model, we tested the two hypothesized interactions. Continuous variables were mean centered prior to creating the interaction terms. These were obtained by multiplying the respective predictor variables. Finally, to analyze whether the relationship between the studied variables and the use of maternal PC varied according to who reports it. The fourth model included cross level interactions. In this model maternal separation anxiety, inter-parental conflict, and both dimensions of adolescents’ temperament were predictors of the relationship between Informant and maternal PC. This fourth model was:


$$\begin{array}{ll} \text{PC = } & \gamma_{\text{00}}\text{+}\gamma_{\text{01}}\times \text{Dyad Type +}\gamma_{\text{02}}\times \text{Adolescent Effortful}\\ & \text{Control +}\gamma_{\text{03}}\times \text{Adolescent Frustration +}\gamma_{\text{04}}\times \text{Maternal}\\ & \text{Anxiety Separation +}\gamma_{\text{05}}\times \text{ Inter-parental Conflict +}\gamma_{\text{06}}\times \\ & \text{Maternal Anxiety Separation }\times \text{ Inter-parental Conflict}\\ & \text{+}\gamma_{\text{07}}\times \text{ Adolescent Effortful Control}\times \text{Frustration +}\gamma_{\text{10}}\\ & \times \text{Informant +}\gamma_{\text{11}}\times \text{Adolescent Effortful Control}\times \\ & \text{Informant +}\gamma_{\text{12}}\times \text{Adolescent Frustration}\times \text{Informant}\\ & \text{+}\gamma_{\text{13}}\times \text{Maternal Anxiety Separation}\times \text{Informant +}\gamma_{\text{14}}\times \\ & \text{Inter-parental Conflict}\times {\text{Informant + u}}_{\text{0}}{\text{ + u}}_{\text{1}}\\ & \times \text{Informant + }r. \end{array}$$

Each model was compared to the previous by deviance difference, and their significance was estimated by Chi-square distribution. When change in deviance exceeds the critical value of Chi-square with degrees of freedom (based on delta of degrees of freedom between models), the difference is statically significant; indicating the more complex model fits better to the data. However, if the more complex model does not result in a statistically significant reduction of the deviance statistic, the more parsimonious model is more adequate. Also, Akaike Informatory Criterion (AIC) and Bayesian Information Criterion (BIC) were estimated from the deviance statistic, by Full Maximum Likelihood estimation method. Models with lowest AIC and BIC are considered to be the fitting model (McCoach and Black, 2008). A final model to account the data was tested and interpreted.

## Results

### Descriptive Analysis and Correlations

Table 1 shows the descriptive results. Regarding the adolescents’ sex, we only observed differences in the level of maternal anxiety about separation from their children, which was greater for daughters (*M* = 3.20, SD = 0.86) than for sons (*M* = 2.76, SD = 0.78), *t*(104) = −2.381, *p* = 0.019. We found no sex differences (*p*_*s*_ > 0.05) in other variables: mother-reported PC [mother–daughter (M-D): *M* = 1.90, SD = 0.85; mother–son (M-S): *M* = 1.58, SD = 0.45]; adolescent-reported PC (M-D: *M* = 1.94, SD = 0.72 and M-S: *M* = 1.92, SD = 0.67); adolescent effortful control (M-D: *M* = 3.18, SD = 0.49 and M-S: *M* = 3.34, SD = 0.43); adolescent frustration control (M-D: *M* = 3.34, SD = 0.59 and M-S: *M* = 3.30, SD = 0.57); and inter-parental conflict (M-D: *M* = 12.73, SD = 9.22 and M-S: *M* = 8.70, SD = 6.91).

**TABLE 1:** Descriptive statistics and correlations.

	*M*	SD	2	3	4	5	6	7	8
1. Adolescent sex (1 = men)	0.77	0.42	−0.10	−0.24*	–0.01	−0.23*	0.15	–0.03	−0.22*
2. Adolescent’ age	15.42	1.09		–0.05	0.09	0.05	–0.07	–0.03	–0.12
3. Psychological control–mother report	1.65	0.58			0.21*	0.37***	−0.34***	0.24*	0.30**
4. Psychological control–adolescent report	1.92	0.68				0.19*	−0.31***	0.27**	0.36***
5. Maternal separation anxiety	2.86	0.81					–0.11	0.06	0.05
6. Effortful control	3.30	0.44						−0.25*	−0.41***
7. Frustration	3.31	0.57							0.04
8. Inter-parental conflict	9.61	7.64							

*N = 106 dyads, *n* = 82 adolescent males and *n* = 24 adolescent females.*

***p < 0.001; **p < 0.01, *p < 0.05.

Considering the bivariate correlations, the PC level reported by both mothers and adolescents positively correlated with maternal separation anxiety, inter-parental conflict, and adolescents’ frustration levels. In addition, we observed a negative correlation with the adolescents’ level of effortful control. The temperamental dimensions – frustration and effortful control – showed a correlation of *r*(104) = −0.25, *p* < 0.05 between them. Finally, the adolescent-reported and mother-reported PC were significantly correlated, *r*(104) = 0.21, *p* < 0.05.

### Hierarchical Linear Regression Models

The Null Model showed that the Intra-Class correlation indicated that 18% of variance of maternal PC is due to between-person differences. A progressive improvement on model adjustment was achieved from the Null Model to Model 2 (Table 2). When Model 3 was considered, deviance difference indicated that this model did not improve significatively the fit, but AIC and BIC showed a small decrease, indicating that this model would better fit the data. In fact, an additional confirmation was done when comparing the previous model with a model that included only the interaction between maternal separation anxiety and conflict, finding a significant improvement in the fit (Δ Deviance = 4.38, 1 *df.*, *p* = 0.034), concordant with the statistical significance of the associated fixed effect. In Model 4, cross-level moderation terms (γ11 to γ14) were not statistically significant, and their AIC, BIC and the Chi-square difference indicator, show that this model does not significantly improve the fit, indicating that the previous model is more adequate to describe the data. Based on these parameters, we conclude that the relations between maternal anxiety about adolescent distancing, inter-parental conflict, and adolescent temperament with the level of maternal PC did not depend on who reported these.

**TABLE 2:** Predictors of psychological control as perceived by mothers and adolescents.

		Null Model		Model 1		Model 2		Model 3		Model 4		Final Model	
		β	Error	β	Error	β	Error	β	Error	β	Error	β	Error
Intercept	γ00	1.79***	0.05	1.92***	0.07	1.92***	0.09	1.91***	0.09	1.91***	0.09	1.91***	0.06
Dyad type (1 = mother-son)	γ01	–	–	–	–	0.01	0.09	0.01	0.09	0.21	0.09	–	–
Adolescent effortful control (EC)	γ02	–	–	–	–	−0.21*	0.09	−0.27**	0.10	–0.24	0.15	−0.26**	0.09
Adolescent frustration (F)	γ03	–	–	–	–	0.21**	0.07	0.19**	0.08	0.25	0.12	0.19**	0.07
Maternal separation anxiety (MSA)	γ04	–	–	–	–	0.19***	0.05	0.20***	0.05	0.16*	0.07	0.20***	0.04
Inter-parental conflict (IPC)	γ05	–	–	–	–	0.02***	0.01	0.02***	0.01	0.03**	0.01	0.02***	0.01
EC × F	γ06	–	–	–	–	–	–	–0.10	0.15	–0.10	0.15	–	–
MSA × IPC	γ07	–	–	–	–	–	–	0.01*	0.01	0.01*	0.01	0.01*	0.01
Informant (1 = mother)	γ10	–	–	−0.27***	0.07	−0.27***	0.08	−0.27***	0.08	−0.27***	0.08	−0.27***	0.08
EC × Informant	γ11	–	–	–	–	–	–	–	–	–0.06	0.21	–	–
F × Informant	γ12	–	–	–	–	–	–	–	–	–0.09	0.14	–	–
MSA × Informant	γ13	–	–	–	–	–	–	–	–	0.11	0.10	–	–
IPC × Informant	γ14	–	–	–	–	–	–	–	–	–0.01	0.01	–	–
Intercept	u0	0.07**		0.18***		0.15 ***		0.16***		0.16***		0.16***	
Informant	u1			0.06		0.20***		0.22***		0.22***		0.22***	
Level-1	*r*	0.35***		0.29***		0.21***		0.21***		0.21**		0.21***	
Deviance^a^		411.58 (3 *df.*)		396.79 (6 *df.*)		340.26 (11 *df.*)		335.44 (13 *df.*)		332.83 (17 *df.*)		335.92 (11 *df.*)	
AIC^b^		417.58		408.79		362.26		361.44		366.83		357.92	
BIC^c^		418.56		410.75		365.85		365.68		372.38		361.51	
Δ Deviance		–		14.79 (3 *df.*)**		56.53 (5 *df.*)***		4.82 (2 *df.*)^£^		2.61 (4 *df.*)		0.48 (2 *df.*)^d^	

*Level-1 *N* = 212; Level-2 *N* = 106. Continuous predictors were mean-centered. Random Effects: variances component and their significance based on Chi-square.^*a*^Deviance and Δ Deviance were based on Full Maximum Likelihood. ^*b*^AIC = Deviance + 2 parameters in the model. ^*c*^BIC = Deviance + ln (*n*) × parameters in the model; being *n* = 212 (Level-1 sample size). ^*d*^Δ Deviance between Final Model vs. Model 3.*

***p < 0.001; **p < 0.01; *p < 0.05; ^£^*p* < 0.09.

**FIGURE 1:**
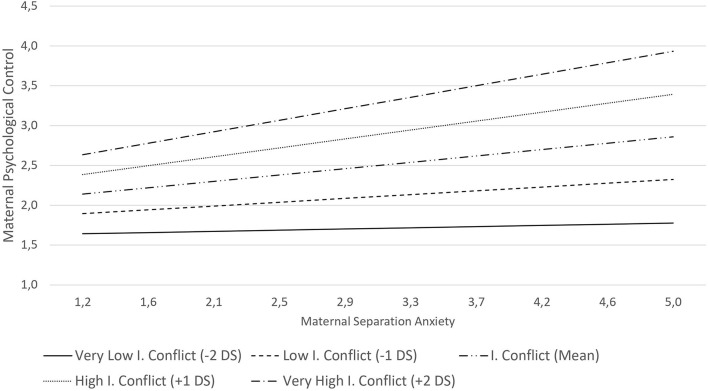
Relations between maternal separation anxiety and maternal psychological control depends on the level of inter-parental (I.) conflicts.

The final model indicates that adolescents reported (*M* = 1.91) higher levels of perceived maternal PC than their mothers (*M* = 1.64). Also, that adolescent frustration is positively correlated with the level of maternal PC as reported by mothers and adolescents and that effortful control is inversely associated with maternal PC. The significant interaction term between maternal separation anxiety and inter-parental conflict (MSA × IPC) indicate that the positive relation between maternal separation anxiety and maternal PC depends on the degree of inter-parental conflict. Specifically, in the case of families with a near-average (9.6 points) inter-parental conflict, there is a positive relationship between maternal separation anxiety and maternal PC (γ04), but this relationship increases by 0.01 (γ07), point as the inter-parental conflict increases in one point (see Figure 1).

## Discussion

Results indicate that adolescents reported higher levels of maternal PC than their mothers and all predictors were associated with maternal PC reports by mothers and adolescents. When maternal anxiety about adolescent distancing, inter-parental conflict, and adolescent frustration were higher, higher levels of PC were reported. Only higher levels of adolescent’s effortful control were associated with lower levels of PC. Finally, results indicate that all variables included were associated with the levels of maternal PC reported by adolescents and their mothers.

The higher levels of maternal PC reported by adolescents (when compared to their mothers’) found in this Latin American sample is consistent with studies conducted in other contexts. A meta-analysis of the congruence of parents’, children’s, and adolescents’ perceptions of parenting dimensions (Korelitz and Garber, 2016) indicated that children of all ages tend to report higher levels of PC than their mothers.

The observation that adolescents report higher levels of maternal PC than their mothers is also consistent with past studies that have shown that parents tend to report more favorably than their children about parenting, parent–child relationships, and family functioning (e.g., Ohannessian et al., 1995; Ohannessian et al., 2000; Ohannessian and De Los Reyes, 2014). For example, adults report being more accepting and/or less controlling than their children report, or perceiving a more cohesive family unit than their adolescent children. In a Chilean sample, Pérez et al. (2018) found that mothers perceived their family as more cohesive and more adaptable than their adolescents. This can be understood in light of the different developmental stages adolescents and their mothers are facing (Bengston and Kuypers, 1971). Specifically, adolescents’ need to achieve autonomy and reduce emotional closeness with their parents (Erikson, 1950; Papalia et al., 2012), and parents need to achieve generativity (Erikson, 1950), might explain the different in perceptions on the quality of parent-adolescent relations.

The study results support our hypothesis and is consistent with previous studies that show a positive relation between maternal anxiety about adolescent distancing and maternal PC (Flett et al., 2002; Soenens et al., 2005; Laird, 2011; Wuyts et al., 2017), and the positive relation between inter-parental conflict and maternal PC (Stone et al., 2002; Buehler et al., 2006; Cabrera et al., 2006).

Regarding the relation between adolescent’s temperamental dimensions and maternal PC, results showed that as levels of frustration were higher or levels of effortful control were lower, higher levels of PC were reported by adolescents and their mothers. Similar findings have been reported by Walling et al. (2007); Laukkanen et al. (2014), and Pérez and Cumsille (2012). These results also support the environmental elicitation phenomenon (Shiner and Caspi, 2003), being the maternal PC higher as their adolescents’ temperamental characteristics fit a difficult temperament or dysregulated profile (low effortful control and/or high frustration).

In fact, the study results showed that multiple conditions could influence family functioning, revealing that maternal and child characteristics, as well as the contextual characteristics are –at least– additive in nature. The characteristics of the different members of the families plays a role in the use of the maternal PC. When facing adolescents with high irritability or mothers with high separation anxiety, a greater use of the maternal PC could be expected. Moreover, the study results showed a significant interaction –or multiplicative effect– between maternal separation anxiety and inter-parental conflict on maternal PC. So, the positive association between maternal separation anxiety and maternal PC will be strengthened in families where interparental conflicts occur.

Different explanations can be proposed to explain this interaction effect. First, the experience of conflict is a highly stressful phenomenon in couples and has negative consequences on the satisfaction and duration of the relationship (Feeney and Karantzas, 2017), thus involving a high level of maternal stress (Sturge-Apple et al., 2003). This stress has been shown to decrease sensitivity in maternal interaction with their young children (Booth et al., 2018), a similar phenomenon could occur in response to their adolescent children’s needs. Thus, this interaction effect could account for a cumulative effect of stressful situations (stress arising from her couple relationship and from their problems to manage the adolescent’s growth and autonomous behavior) that cannot be managed properly by the mother, resorting to PC to reduce her own anxiety (Lyons-Ruth and Jacobvitz, 2016).

Another plausible explanation of this interaction effect would be that it is indicative of the personal characteristics of the mother, in lines to Barber et al. (2002) proposal, that the parent’s own psychological status impacts the use of PC. Thus, mothers who present a high level of neuroticism, characterized by a proneness to psychological distress, unrealistic ideas, excessive cravings, and maladaptive coping responses (Bornstein et al., 2011), would be more susceptible to experience separation anxiety and –at the same time– get more frequently involved in conflicts with their partner. So, mothers having high separation anxiety and high interparental conflict, will be more prone to use PC. In fact, multiple studies have shown that maternal neuroticism is correlated with intrusiveness, irritability, criticism, negative discipline, hostility, and power assertion toward their children (Bornstein et al., 2011; Coplan et al., 2009).

Finally, a secondary result indicates that maternal separation anxiety differed according to adolescent’s gender, being greater for daughters than sons. This finding could be explained by the cultural characteristics of the sample, since traditional gender roles are still predominating in Latin America and Chile, particularly in those women who exercise care roles (PNUD, 2019). Therefore, mothers would exert more control over their daughters than their sons, due to the preconceptions of weakness attributed to women and the need for greater protection associated to their sexual development and couple formation (Asociación Chilena Pro Naciones Unidas, ACHNU—Prodeni, 2006; Jiménez-Moya et al., 2020).

Of interest to clinical psychologists, the present findings suggest that different paths can be taken to decrease the use of maternal PC. For example, interventions aimed at improving the capacities for emotional regulation and adequate coping with stress for both adults and adolescents could act as protective factors in the use of this parental control strategy. Likewise, couples therapy, by reducing the frequency and intensity of inter-parental conflicts, could reduce the use of maternal PC.

In fact, future research could study the potential mediating role of stress sensitivity and emotional regulation capacities in the relationship between the identified variables and the use of maternal PC, in order to confirm the usefulness of the previously proposed strategies. It would also be relevant to develop studies based on a methodology centered on the person (and not on variables), to determine which cluster of maternal characteristics are related to a greater use of PC.

Although this study contributes to the literature by examining the association between certain predictors and maternal PC, the results from the present study should be considered in light of some limitations. Because of the cross-sectional nature of the design, it is not possible to establish the causality of the effects of these predictors on maternal PC. Future longitudinal studies could address this issue to clarify the nature of these relations. A second limitation concerns the exclusive inclusion of mothers in the present study, as fathers’ characteristics may have a significant influence on family functioning or buffer the effects reported in the present study. Third, this study relies solely on self-report measures and interparental conflict was not measured by objective instruments (i.e., direct observation of the parent’s interaction). Finally, the nature of the sample presents limitations, on one hand, male adolescents are over represented. Also, the sample size places limits for the estimation of higher-order interactions, since it does not have the necessary power for such analyzes.

Despite these limitations, the present study complements previous findings by demonstrating how the child’s characteristics, mother’s characteristics and contextual factors are associated with maternal PC, not just in an additive but also in multiplicative form, helping to understand how these conditions could increase the use of maternal PC. These perspectives on multiple conditions can also be a potential source of change, as it might allow to modify the use of maternal PC by carrying out interventions, in the different actors of the family system, even in those not included in the present study (i.e., by reducing intra-parental conflicts). Also, this study was developed in a scarcely studied context, by including Latin American adolescents and their mothers, contributing to the cross-cultural study of maternal PC. Finally, it should be noted that these results were obtained using a multi-informant perspective and a statistical method that considers the relation between different reports of maternal PC (by adolescents and by mothers).

## Data Availability Statement

The raw data supporting the conclusions of this article will be made available by the principal author, without undue reservation.

## Ethics Statement

The studies involving human participants were reviewed and approved by the Ethics Committee of the Universidad del Desarrollo. Written informed consent to participate in this study was provided by the participants’ legal guardian/next of kin. Also, adolescents signed an informed assent.

## Author Contributions

JP, PH, and BR contributed to conception and design of the study. JP got the funding resources and performed the statistical analysis. PH and BR obtained the data. JP and OF wrote the draft of the manuscript. All authors contributed to manuscript revision, read, and approved the submitted version.

## Conflict of Interest

The authors declare that the research was conducted in the absence of any commercial or financial relationships that could be construed as a potential conflict of interest.

## Publisher’s Note

All claims expressed in this article are solely those of the authors and do not necessarily represent those of their affiliated organizations, or those of the publisher, the editors and the reviewers. Any product that may be evaluated in this article, or claim that may be made by its manufacturer, is not guaranteed or endorsed by the publisher.
