# Corrigendum: Parental psychological control: Maternal, adolescent, and contextual predictors

**DOI:** 10.3389/fpsyg.2022.979872

**Published:** 2022-07-29

**Authors:** J. Carola Pérez, Paula Huerta, Bernardita Rubio, Olga Fernández

**Affiliations:** ^1^Centro de Apego y Regulación Emocional, Universidad del Desarrollo, Santiago, Chile; ^2^Master's Program in Adolescent Psychology, Universidad del Desarrollo, Santiago, Chile; ^3^Psychiatry and Mental Health Department, Faculty of Medicine, Universidad de Chile, Santiago, Chile

**Keywords:** maternal psychological control, adolescent irritability, adolescent effortful control, maternal separation anxiety, inter-parental conflict

In the published article, there was an error in the Funding statement. One of the grants of “Fondo Nacional de Desarrollo Científico y Tecnológico, Chile” (FONDECYT) was displayed as “1201675.” The correct Funding statement appears below.

## Funding

This study was funded by grant Nos. 11130041 and 1201576 from “Fondo Nacional de Desarrollo Científico y Tecnológico, Chile” (FONDECYT) and supported by ANID Millennium Science Initiative/Millennium Institute for Research on Depression and Personality-MIDAP ICS13_005.

The authors apologize for this error and state that this does not change the scientific conclusions of the article in any way. The original article has been updated.

## Publisher's note

All claims expressed in this article are solely those of the authors and do not necessarily represent those of their affiliated organizations, or those of the publisher, the editors and the reviewers. Any product that may be evaluated in this article, or claim that may be made by its manufacturer, is not guaranteed or endorsed by the publisher.

