# Supplementary material for: Parental Psychological Control: Maternal, Adolescent, and Contextual Predictors
Source: Front Psychol. 2021 Sep 21;12:712087. doi: 10.3389/fpsyg.2021.712087 (PMC8490725; doi:10.3389/fpsyg.2021.712087)
Supplement: Supplementary file 1 [file Table_1.DOCX]

Supplementary Material

# Table 1. Factorial Invariance of Psychological Control – Disrespect Scale

| Models | *df* | *X*^2^ | RMSEA | (90% CI) | CFI | Δ *X*^2^ (*gl*.) | ΔCFI | |
| --- | --- | --- | --- | --- | --- | --- | --- | --- |
| Estimated models according to who reports^a^ | | | | | | | |  |
| Adolescents | 14 | 25.900* | .090 | .030-.143 | .869 |  |  | |
| Mothers | 14 | 16.258 | .039 | .000-.106 | .984 |  |  | |
| Invariance | | | | | | | |  |
| Configural | 28 | 40.205 | .064 | .000-.106 | .947 |  |  | |
| Metric | 34 | 43.128 | .050 | .000-.091 | .960 | 2.418 (6 *gl*.)^b^ | 0.013 | |
| Scalar | 41 | 66.264** | .076 | .040-.109 | .891 | 23.986** (7 *gl*.)^c^ | -0.069 | |
| Partial Scalar^d^ | 39 | 52.670 | .058 | .000-.094 | .941 | 9.693 (5 *gl*.)^e^ | -0.019 | |

*Note*. *df* = degrees of freedom; χ2= Chi-Square; RMSEA = root mean square error of approximation; 90% CI = 90% confidence interval for the RMSEA; CFI = comparative fit index. The Δ index comparison was made with respect to the configural model. ^a^One-factor solution which includes seven of the eight original items. ^b^Delta of chi-square were estimated rescaling this value by correction factor (1.2324 for configural model & 1.2374 for metric invariance); ^c^Delta of chi-square were estimated rescaling this value by correction factor (1.1880 for scalar model) and comparison model was metric invariance; ^d^Item intercept for item 2 and item 7 were not constrained. ^b^Delta of chi-square were estimated rescaling this value by correction factor (1.2020 for partial scalar model) and comparison model was metric invariance.

** *p* < .01; * *p* < .05

Table 2. Factor solution of Parents of Adolescents Separation Anxiety Scale (PASAS)

|  | Factors | | |
| --- | --- | --- | --- |
|  | Maternal Separation  Anxiety | Discomfort with Secure Base Role | Comfort with Secure Base Role |
| Item 18 | .707 |  |  |
| Item 15 | .691 |  |  |
| Item 31 | .680 |  |  |
| Item 17 | .678 |  |  |
| Item 12 | .615 |  |  |
| Item 7 | .597 |  |  |
| Item 16 | .580 |  |  |
| Item 24 | .578 |  |  |
| Item 22 | .571 |  |  |
| Item 28 | .530 |  |  |
| Item 14 | .516 |  |  |
| Item 29 | .504 | .303 |  |
| Item 26 | .487 |  |  |
| Item 30 | .482 |  |  |
| Item 11^a^ | .479 |  |  |
| Item 13 | .467 |  |  |
| Item 9 | .398 |  |  |
| Item 32 | .391 |  |  |
| Item 34 | .384 |  |  |
| Item 19 | .339 |  |  |
| Item 8 | .313 |  |  |
| Item 6 | .303 |  |  |
| Item 5 |  | .677 |  |
| Item 35 |  | .664 |  |
| Item 10 |  | .508 | .471 |
| Item 25 |  | -.398 |  |
| Item 27 |  | .355 | .328 |
| Item 23 |  |  | .556 |
| Item 33 |  | -.311 | .508 |
| Item 21 |  |  | .387 |
| Item 2 |  |  | .372 |
| *Note*. Principal Axis Factoring. No rotation. Chilean version of item 11 is “It bothers me if my teenager keeps secrets about himself/herself from me”. All loading factors < .30 were not included. | | | |
